# Tribocorrosion Susceptibility and Mechanical Characteristics of As-Received and Long-Term In-Vivo Aged Nickel-Titanium and Stainless-Steel Archwires

**DOI:** 10.3390/ma15041427

**Published:** 2022-02-15

**Authors:** Jasmina Primozic, Miha Hren, Uros Mezeg, Andraz Legat

**Affiliations:** 1 Faculty of Medicine, University of Ljubljana, SI-1000 Ljubljana, Slovenia; beli.medved@telemach.net; 2 Slovenian National Building and Civil Engineering Institute, SI-1000 Ljubljana, Slovenia; miha.hren@zag.si (M.H.); andraz.legat@zag.si (A.L.)

**Keywords:** tribocorrosion, orthodontic archwires, electrochemical noise

## Abstract

To evaluate the effect of long-term in-vivo aging on orthodontic archwires, we aimed to assess the triboelectrochemical and mechanical characteristics of as-received and in-vivo aged nickel-titanium (NiTi) and stainless-steel (SS) orthodontic archwires. Four consecutive tribocorrosion cycles on six NiTi and six SS archwires, as-received and in-vivo aged, were performed on a reciprocal tribometer. Electrochemical noise and friction coefficient measurements, three-dimensional surface profiling, and hardness measurements were performed. Repassivation times of as-received archwires were longer than of the in-vivo aged; however, were shorter for NiTi. Friction coefficients were higher for NiTi than for SS archwires. Sudden major current drops concomitant with inverse potential shifts and friction coefficients’ fluctuations, were seen for as-received (last cycle) and in-vivo aged (last three cycles) NiTi archwires. More pronounced tribocorrosion damage was observed on in-vivo aged NiTi than on other archwires. Hardness was generally higher inside the wear track of archwires. Long-term in-vivo exposure decreases the corrosion susceptibility of archwires, more evidently for the NiTi ones. Sudden major fluctuations in electrochemical current, potential, and friction coefficient detected for NiTi archwires, might be related to localized residual parts of the oxide layer persisting due to increased surface roughness or to phase transformations of the alloy’s crystal structure.

## 1. Introduction

In dentistry, a wide variety of alloys have to resist the combined action of corrosion and wear in the oral cavity [1]. Briefly, alloys for implants, prostheses, and orthodontic appliances in the oral environment have to resist corrosion (i.e., due to the low intraoral pH) [2] and mechanical loading due to chewing [3]. However, orthodontic appliances are also subjected to further mechanical stresses because of their specific use [4]. Indeed, the sliding of orthodontic archwires along the bracket’s slot can lead to additional and accelerated mechanical wear, as it might destroy the protective surface oxide layer, exposing the metal to the corrosive environment [4]. Depending on the metal alloy [5], the electrolyte composition [6,7], and the loading conditions [8], either the formation of a new oxide layer (repassivation) or the local dissolution of the metal can occur. Thus, the interaction between corrosion and wear is important since wear can break up and remove the protective oxide layer, thereby exposing the metal to corrosion [9,10]. Accelerated material loss is influenced by both corrosion and wear and depends on the characteristics of the oxide layer (i.e., stability), its formation/disruption kinetics (i.e., repassivation velocity), and the properties of the metal (i.e., hardness) [11].

Although various alloys have been developed for orthodontic purposes, during sliding mechanics, the most frequently used archwires are those made of stainless steel (SS) alloys (mainly AISI 302 and 304) because of their low coefficient of friction, which decreases the resistance to sliding of the archwire [12]. Moreover, several nearly equiatomic nickel-titanium (NiTi) alloys are also widely used in orthodontics due to their valuable characteristics, such as shape memory and pseudo-elasticity. The shape-memory effect and pseudo-elasticity rely on the presence of austenite-martensite phase transformations that are induced by either temperature changes or the application of stress, respectively [13].

The corrosion properties of orthodontic archwires have been widely studied because of the associated biocompatibility issues [14,15,16,17,18,19,20], whereas very little information is available regarding their tribocorrosion properties [9,21,22,23]. In particular, mainly as-received orthodontic archwires have been studied [21,22,23]. Močnik et al. [23] reported the tribocorrosion effects on as-received NiTi and SS archwires and concluded that the tribological contact in artificial saliva did not significantly affect the structure of the oxide layer. A recent study [9] reported that in-vivo exposed NiTi and SS archwires had better electrochemical properties than new archwires due to the protective nature of oral deposits. On the other hand, a higher risk of material breakdown has been reported [24,25,26] after exposure in the oral environment, therefore it appears that long-term exposure to corrosion and mechanical wear might affect the mechanical properties of archwires (i.e., coefficient of friction, hardness, etc.), and because of that, their performance.

Among the different experimental approaches that can be used to assess material tribocorrosion properties [27], electrochemical noise measurements can be used to monitor transient corrosion events. Briefly, since electrochemical noise consists of current and potential fluctuations spontaneously generated by corrosion processes, no external electrochemical intrusion is needed. In corrosive wear systems, the electrochemical current noise is measured between two originally identical working electrodes, of which one is tribologically disturbed, while the electrochemical potential noise is usually measured between the coupled metal electrodes and the reference electrode [28].

Despite a high number of studies on corrosion of orthodontic archwires, to the best of our knowledge, there is still a paucity of data regarding the tribocorrosion susceptibility of NiTi and SS archwires after their long-term use in the oral environment. In particular, there is a lack of studies using a combination of electrochemical (electrochemical noise) and mechanical (coefficient of friction) measurements for comparisons of as-received and in-vivo aged orthodontic archwires. Furthermore, scarce information is available about how continuous tribocorrosion affects the mechanical properties of dental alloys, including the hardness, surface roughness, and coefficient of friction, and therefore what impact it has on their long-term clinical performance. In fact, tests performed on as-received and/or in-vitro aged material might not completely account for the long-term intraoral aging of dental alloys.

To evaluate the effect of long-term in-vivo aging on orthodontic archwires, the present study aimed to assess the triboelectrochemical and mechanical characteristics of as-received and in-vivo aged NiTi and SS archwires. The relationship between electrochemical and mechanical parameters was determined, and these parameters were related to the type of archwires and the actual damage occurring on them.

## 2. Materials and Methods

### 2.1. Specimen Preparation

Specimens for the triboelectrochemical testing were prepared from as-received and in-vivo aged rectangular 0.457 mm × 0.635 mm NiTi (Neo Sentalloy; 51% Ni, Ti balance) and SS (AISI 304 alloy; 69.5% Fe, 18.5% Cr, 9% Ni, 1% Mn, 0.75% Co, Si, C, P and S less than 1%) archwires (Dentsply GAC, New York, NY, USA). In-vivo aging was performed for eight weeks in the oral environment of the same subject, with each archwire engaged in the bracket’s slots (made of AISI 316 SS) with elastomeric ligatures. Ethical approval of the national ethical committee (Ref.126/04/13) and informed consent of the included subject were gained before the beginning of the study.

**Figure 1:**
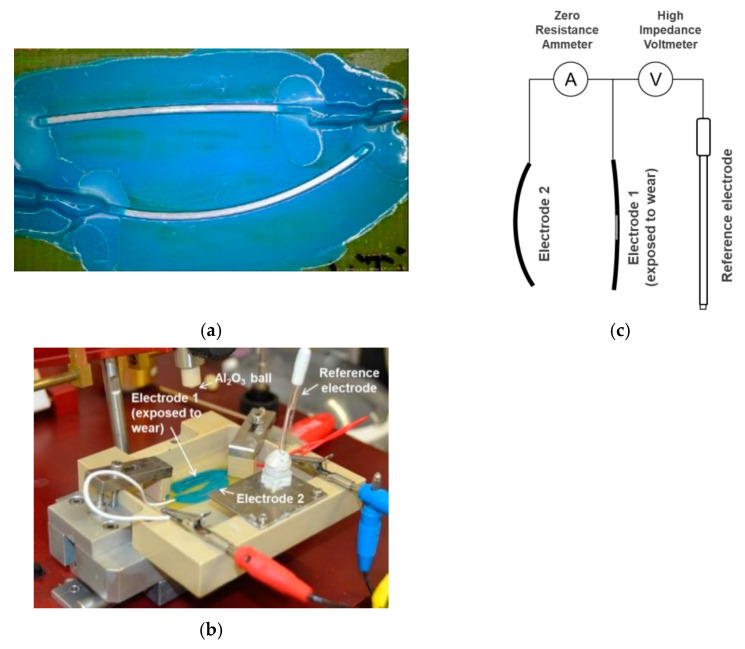
Experimental setup: (**a**) preparation of specimen with the straight (electrode exposed to wear) and curved (counting electrode) part of the archwire, glued to a glass-reinforced epoxy laminate sheet; (**b**) experimental setup with important parts highlighted; (**c**) schematic representation of the electrochemical noise measurement setup.

Each specimen (Figure 1a) consisted of two electrodes cut from the same archwire. The straight part of the archwire (approximately 30 mm) was used as the electrode exposed to wear, while the curved part was used as the counter electrode. Copper electrical wires were soldered at one end of each archwire with Sn63Pb37 and shielded from exposure using multiple protective coatings (Figure 1b). Both archwires of each specimen were glued to a glass-reinforced epoxy laminate sheet with cyanoacrylate glue. Each specimen was connected to a high-impedance voltmeter and a zero-resistance ammeter, both integrated into a single specialized device. The voltmeter was connected to the counter electrode on one end and to the electrode exposed to wear on the other (Figure 1c), while an Ag/AgCl electrode was used as a reference. A total of twelve specimens, six NiTi (three as received and three in-vivo aged) and six SS (three as received and three in-vivo aged) archwires, were retrieved.

### 2.2. Triboelectrochemical Experiments

Each specimen was exposed to mechanical wear and a solution of artificial saliva according to Duffo and Castillo [29] consisting of 0.60 g/L NaCl, 0.72 g/L KCl, 0.22 g/L CaCl_2_·2 H_2_O, 0.68 g/L KH_2_PO_4_, 0.856 g/L Na_2_HPO_4_·12 H_2_O, 0.060 g/L KSCN, 1.5 g/L KHCO_3_, and 0.03 g/L citric acid and a pH of 6.5. The tribocorrosion testing was performed on a reciprocal tribometer (a pin-on-disc and reciprocating tribometer, Tribotechnic, 2009, France) with a grade-10 ceramic Al_2_O_3_ ball according to the ISO 3290 standard as a counter body.

The whole specimen was fully immersed in artificial saliva during exposure, whereas the mechanical wear on one electrode was conducted in cycles. The cyclic tribocorrosion exposure lasted for roughly 13 h, with approximately 11 h breaks in between. This was repeated four times for four consecutive days (cycles) of exposure. The artificial saliva was replaced with a fresh batch every day before each wear cycle. The measuring equipment was turned off during this time, and electrodes were exposed to air for approximately 1 min.

A vertical force of 1 N was applied during the 13 h tribocorrosion cycles. The length of the wear track was 5 mm. The speed of the ball was set to 0.6 mm/s, which meant that one wear path was completed in roughly 9.8 s. It should be mentioned that the speed approximately followed a sinusoidal function, but the maximum value was not known precisely. The sampling frequency was calculated based on the total exposure time and was roughly 0.2 Hz for all the specimens. 

Both the potential and current measurements were sampled with a frequency of 10 Hz during the electrochemical noise measurements. The signal frequencies above 5 Hz were filtered. The upper and lower limit for the potential measurement was set at ±1 V, while the current limit was set at ±10 µA. In both cases, a 16-bit A/D converter was used within these boundaries. The corrosion current was measured between the archwires of each specimen. Connections to the electrodes ensured that the positive current represented the anodic behavior of the electrode exposed to tribocorrosion. As the ball cyclically moved across the 5 mm path, the coefficient of friction was measured. Within each cycle, mean and standard error with a 95% confidence interval of repassivation times, electrochemical potentials, currents, and friction coefficients were calculated based on the measurements on three specimens of each as-received or in-vivo aged NiTi or SS archwires.

### 2.3. Surface Profiles, Roughness, and Hardness Measurements

An optical microscope (Tagarno A/S, Horsens, Denmark) at a magnification of 105× was used to examine the surface of specimens and ceramic ball before and after testing. Three-dimensional surface profiles of all the specimens were measured using a Form Talysurf Series 2 (Taylor-Hobson, Leicester, UK). The surface was profiled on one end of the electrode exposed to the wear. The hardness was measured on the electrodes exposed to the wear, both inside and outside of the exposed area. The Vickers hardness tests were performed using an MVK-H2 Hardness tester (Mitutoyo Europe Gmbh, Neuss, Germany), applying a load of 1 N. Moreover, a scanning electron microscope (SEM; JEOL JSM-IT500) with an energy-dispersive X-ray spectroscopy (EDS) analyzer (Oxford Instruments) was used for the surface analysis and the determination of the chemical composition of as-received and in-vivo aged archwires after the triboelectrochemical experiments. Analysis was performed on the archwire used as the working electrode at different locations: inside the wear track, outside it, and at the edge of the wear track. The archwires used as the counter electrode was also analyzed. The chemical composition was expressed in weight percentages.

## 3. Results

### 3.1. Triboelectrochemical Properties

**Figure 2:**
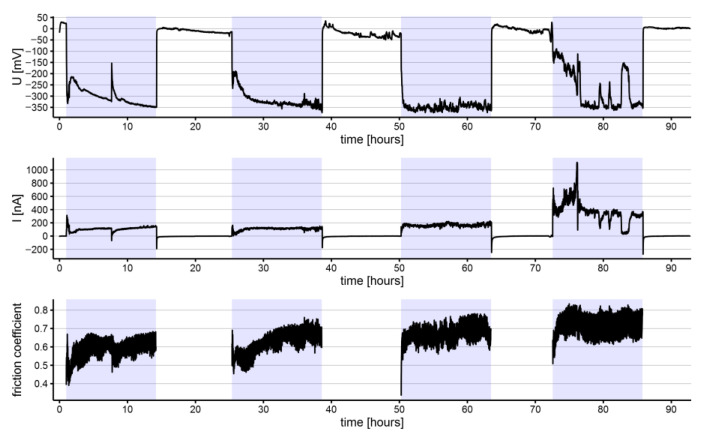
Electrochemical potential (U), current (I), and coefficient of friction measured on an as-received NiTi archwire (highlighted sections represent intervals where the specimen was exposed to tribocorrosion cycles).

**Figure 3:**
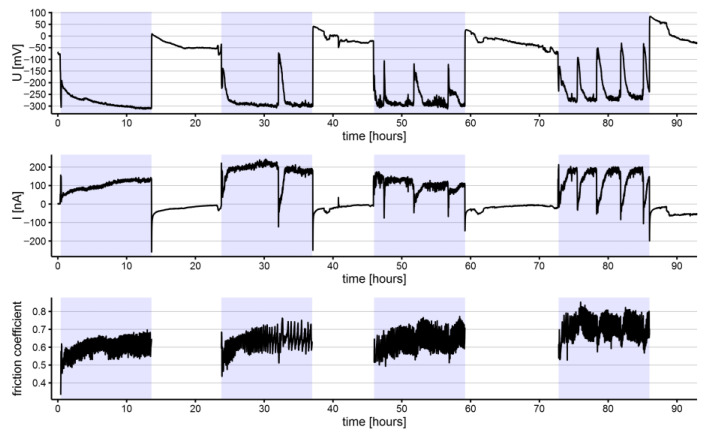
Electrochemical potential (U), current (I), and coefficient of friction measured on an in-vivo aged NiTi archwire (highlighted sections represent intervals where the specimen was exposed to tribocorrosion cycles).

**Figure 4:**
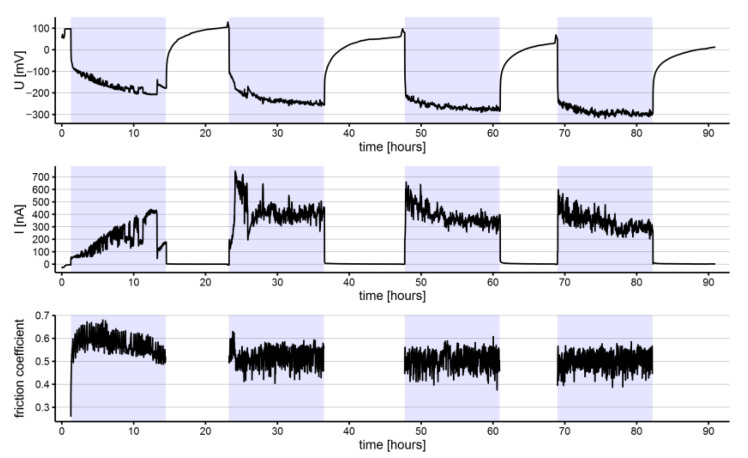
Electrochemical potential (U), current (I), and coefficient of friction measured on an as-received SS archwires (highlighted sections represent intervals where the specimen was exposed to tribocorrosion cycles).

**Figure 5:**
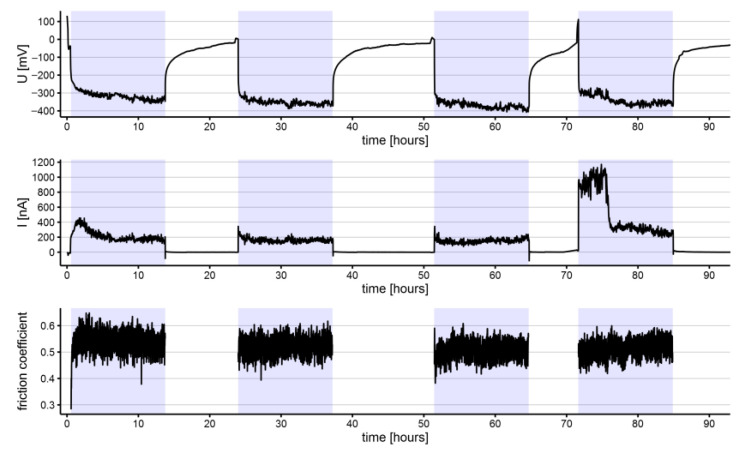
Electrochemical potential (U), current (I), and coefficient of friction measured on an in-vivo aged SS archwire (highlighted sections represent intervals where the specimen was exposed to tribocorrosion cycles).

The electrochemical noise (potential and current) and coefficient of friction measurements for representative specimens of NiTi and SS archwires are shown in Figure 2, Figure 3, Figure 4 and Figure 5. All the shown signals represent the average of measured values within intervals of 100 s, reducing the quantity of recorded data. Within each group of archwires, either NiTi or SS, as-received or in-vivo aged, differences between recorded signals upon repeated measurements do exist; however, for comparisons, average values of the three measurements of each parameter (repassivation time, electrochemical potential, current, and coefficient of friction) were used and are presented along with their scatter in Table 1. Figures of all three measurements according to the type and aging of the archwire are reported as Supplemental Materials.

Average repassivation times among the four cycles for the NiTi archwires were generally shorter than those of the SS archwires. The average repassivation time of as-received NiTi archwires was longer than in-vivo aged NiTi archwires (73.3 ± 16.2 min and 7.4 ± 1.7 min, respectively). In contrast, the repassivation time of as-received and in-vivo aged SS archwires was similar (372.7 ± 66.0 min and 275.5 ± 75.0 min, respectively). Of note, the average repassivation times were shorter for in-vivo aged material, significantly for NiTi archwires. It can be noticed that there were relatively high scatters between the repassivation times within the three specimens of each as-received or in-vivo aged type of archwire (NiTi or SS). A relatively low scatter of the repassivation times was observed only for the in-vivo aged NiTi archwires. 

The average electrochemical potentials among the four cycles measured on the different types of archwires were generally similar. However, quite a substantial scatter between the measurements within each experimental group was observed: this scatter was particularly high for the as-received NiTi and the in-vivo aged SS wires. The initial electrochemical conditions for these two types of archwires varied significantly. 

The average anodic currents generated by the tribocorrosion process were lower for the NiTi archwires than for the SS archwires, confirming higher corrosion activity on the SS archwires (Table 1). Similarly, as for the electrochemical potentials, the scatter was the highest for the as-received NiTi and the in-vivo aged SS archwires, whereas, for the in-vivo aged NiTi archwires and as-received SS archwires, it was substantially lower. Roughly, the lowest electrochemical currents were measured for all the archwires in the first wear tribocorrosion cycle, except for as-received NiTi archwires. The lowest current was measured for the in-vivo aged NiTi archwires (74 ± 28 nA) and the highest for the as-received SS archwires (261 ± 79 nA). During the fourth tribocorrosion cycle; however, an evident increase in the electrochemical current was observed for the as-received NiTi (175 ± 197 nA) and the in-vivo aged SS archwires (312 ± 261 nA), compared to the initial measurements. 

Of note, specific major fluctuations of the electrochemical parameters were recorded for the NiTi archwires. Such spikes of electrochemical potential and current appeared during the third and fourth tribocorrosion cycles for as-received NiTi archwire (Figure 2) and during the second, third, and fourth cycles for the in-vivo aged NiTi archwire (Figure 3). The negative current drops coincided with positive electrochemical potential fluctuations in all cases. The rate of occurrence of these fluctuations generally increased with the number of tribo-cycles. Based on the measured time intervals between the fluctuations, it is evident that these fluctuations were not periodic since different time intervals were recorded between them. On as received and in-vivo aged SS archwires, no similar electrochemical current and potential fluctuations were observed.

The average coefficients of friction were slightly higher for the NiTi (0.66 ± 0.01 and 0.64 ± 0.02 for as-received and in-vivo aged, respectively) than for the SS (0.55 ± 0.02 and 0.55 ± 0.01 for as-received and in-vivo aged, respectively) archwires. Through the tribocorrosion cycles, the average coefficients of friction increased for the NiTi archwires (Table 1), both for the as-received and the in-vivo aged, and in-vivo aged SS archwires, whereas a decreasing tendency was observed among as-received SS archwires over the tribo-cycles. On the NiTi archwires, specific fluctuations of the friction coefficient were observed. These fluctuations were synchronized with the previously mentioned low-frequency major fluctuations of potential and current.

### 3.2. Surface Profiles and Hardness

**Figure 6:**
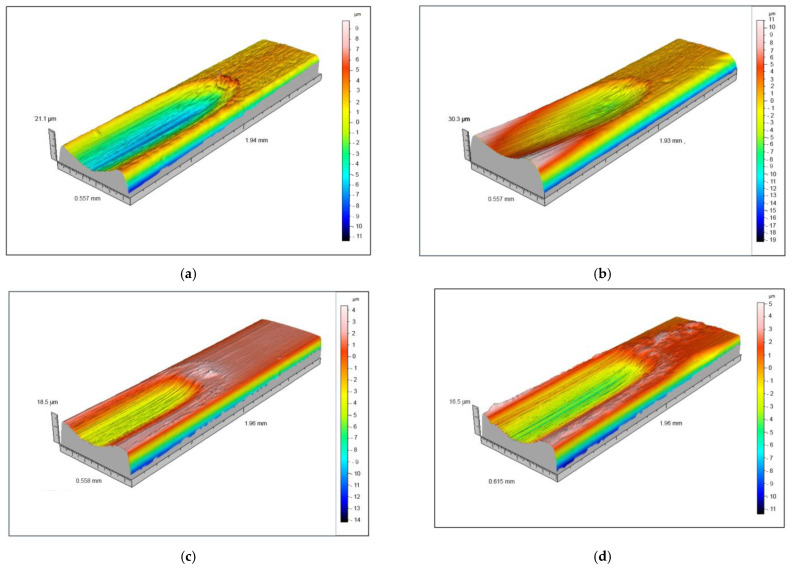
Three-dimensional surface profiles of archwires exposed to tribocorrosion: (**a**) as-received NiTi, (**b**) in-vivo aged NiTi, (**c**) as-received SS, and (**d**) in-vivo aged SS (note: the vertical scales are not identical for each archwire).

The three-dimensional profiles of as-received and in-vivo aged NiTi and SS archwires after tribocorrosion experiments are shown in Figure 6. The profiles of the archwires were generally comparable, but specific differences existed. The greatest depth (between 20 and 30 μm) was measured for the in-vivo aged NiTi archwire (Figure 6b), whereas the depths on the other archwires were roughly similar (between 5 μm and 10 μm). It can also be seen that the longitudinal profiles of the damage on these three archwires (as-received NiTi, as-received, and in-vivo aged SS) are generally symmetrical, while the profile on the in-vivo aged NiTi archwire is uneven. Due to the limited spatial resolution, it was impossible to quantify the perpendicular profiles. However, longitudinal scratches were somewhat more visible on the SS archwires (Figure 6c,d) than on the NiTi archwires (Figure 6a,b). Hence, the profiles of the tribocorrosion tracks on the NiTi archwires are slightly smoother than those of the SS archwires. In addition, the profiles on the as-received archwires appear rougher than those on the in-vivo aged archwires.

The measured roughness and hardness outside and inside the wear track for every specimen are reported in Table 2. The greatest roughness outside the wear track was measured for the in-vivo aged SS archwire, while the smallest was observed in the as-received SS archwire. Lower roughness was observed outside the wear track for the in-vivo aged NiTi archwire than for the as-received one. The NiTi archwires had higher roughness inside the wear track than the SS archwires. In general, the roughness was lower inside than outside the wear track for the NiTi archwires. While lower roughness was measured for the SS archwires, particularly for in-vivo aged SS archwires in which roughness inside the wear track was substantially lower than that measured outside of it. Hardness was generally higher inside the wear track than outside it, except the in-vivo aged SS archwire, where the measured hardness (along with the scatter) was higher outside the wear track.

SEM and EDS analysis revealed certain differences between areas inside and outside the wear track of all types of archwires. The highest oxygen content was detected on the edge of the wear track in all types of archwires as a consequence of agglomeration of worn particles. For NiTi archwires, the oxygen weight percentage was higher inside the wear track than outside it on both as-received and in-vivo aged archwires. The counter NiTi electrode exhibited a similar percentage of oxygen as the exposed electrode outside the wear track. In the area of agglomerated worn particles, other elements were detected, including P, K, and Ca on both as-received and in-vivo aged NiTi archwires, while Na and Si were detected only on in-vivo aged material. The slightly higher oxygen content inside than outside the wear track confirms the fast repassivation capability of NiTi archwires, both as-received and in-vivo aged during the triboelectrochemical experiment. On the contrary, on SS archwires, the EDS results showed slightly lower oxygen weight percentages inside than outside the wear track. Moreover, this phenomena was more evident on in-vivo aged than as-received SS archwires, indicating a thinner passive film on SS archwires. In the areas of agglomerated worn particles, a wide range elements (Na, P, K, Ca, Cl) of typical saliva origin was detected also on SS archwires. The specimen figures obtained using SEM and the chemical composition of selected areas analyzed with EDS are reported in the Supplemental Materials. 

## 4. Discussion

The present study aimed to compare the susceptibility to corrosion and wear of NiTi and SS orthodontic archwires and to assess eventual changes of their characteristics during clinical use. Since orthodontic archwires are subjected to many different biological, chemical, and mechanical factors affecting their surface properties in the oral cavity, it is challenging to reproduce similar in-vitro aging. Therefore, in-vivo aging of each archwire was performed for eight weeks, which approximates the usual clinical time of their intraoral exposure.

Through the triboelectrochemical experiments, the removal of the oxide layer on the as-received and in-vivo aged archwires was performed during four consecutive cycles. The parameters (i.e., force applied, speed of the ball and length of the wear track) used for the triboelectrochemical experiments were based on a preliminary study [22], while the wear cycles interrupted by periods of rest were intended to mimic the clinical condition, since force is usually not constantly applied on archwires. Obviously, to more accurately represent the clinical circumstances, it would have been better to perform the experiment over a longer period of time. However, because of the complexity of the experiment, in particular the need to maintain very stable conditions over the sampling time, four cycles with accelerated wear interrupted by breaks without wear were used here-in. Of particular note, on the in-vivo aged archwires, the oxide layer was removed together with the debris that accumulated during their intraoral exposure. The rate of the oxide layer’s recovery after the tribocorrosion process is indicated by the return of the electrochemical potential to the initial value, which is defined as the repassivation time [4,30,31]. As expected, the repassivation time measured during the experiments was substantially shorter for the NiTi than SS archwires. Interestingly, both in-vivo aged archwires had shorter repassivation times than the respective as-received archwires. This was correlated with the oxygen weight percentages inside the wear track of the examined archwires, indicating the presence of an oxide surface layer.

The anodic currents generated by the tribocorrosion process were lower for the NiTi archwires than for the SS archwires, confirming the higher corrosion activity on the SS archwires. It should be mentioned that within each archwire material type, lower anodic currents were generated on in-vivo aged compared to as-received archwires. However, a certain scatter between the electrochemical responses was observed, and it was the highest for the as-received NiTi archwires and the in-vivo aged SS archwires, whereas, for the in-vivo aged NiTi archwires and the as-received SS archwires, it was substantially lower.

It is believed that these differences originated predominantly from different initial surface conditions of the archwires. Although the specimens could not be taken from the exact locations of the archwires and the initial surface conditions were not precisely equal, based on the measured electrochemical potentials and anodic currents, it might be concluded that in-vivo aging decreased both the repassivation time and the measured anodic currents on NiTi and SS archwires, reducing their corrosion susceptibility. However, despite the scatter, this effect of in-vivo aging was more pronounced on NiTi than SS archwires.

It is yet not clear whether apposition of debris or changes of the mechanical characteristics (i.e., plastic deformation during intraoral exposure) of the archwires due to in-vivo aging influenced the measured repassivation times and currents. It is possible that debris deposits protected the surface of in-vivo aged archwires; however, distinctively according to archwire material. Presumably, debris on in-vivo aged NiTi archwires was formed relatively uniformly and in a thinner layer, while on the in-vivo aged SS wires fairly non-homogeneously with different thicknesses and compactness along the wire. This hypothesis is in agreement with the roughness measurements outside the wear track area, where lower roughness was measured for the in-vivo aged NiTi archwires than the as-received ones, while the opposite was seen for in-vivo aged SS archwires.

Along with the velocity of the oxide film formation, changes in the mechanical characteristic of archwires during their clinical use might also affect their corrosion susceptibility. Comparisons of hardness, which were generally higher inside the wear track than outside it, would confirm the tribological process-induced plastic deformation of the metals. In fact, during the triboelectrochemical experiments, either elastic or plastic deformation of the archwire could occur, exposing the surface of the archwire to corrosion one to three times due to elastic and one to five times due to plastic deformation [4]. It is plausible that during in-vivo exposure, elastic deformation of the NiTi and plastic deformation of the SS archwires more frequently occur, leading to a higher susceptibility to corrosion of SS than the NiTi archwires.

Frequent electrochemical current and potential fluctuations were in complete agreement with the sliding of the ceramic ball that caused rapid corrosion initiation and repassivation. A comprehensive explanation of these fluctuations was not among the goals of the present study, but it is believed that electrochemical modeling using various equivalent electrical circuits would help describe the involved processes [32]. However, sudden major fluctuations were detected during the triboelectrochemical experiments on the NiTi archwires. These major fluctuations were more frequent on the in-vivo aged than the as-received NiTi archwires. Their first appearance was already during the second rubbing cycle of the in-vivo aged NiTi archwires, whereas for the as-received NiTi archwires, major fluctuations were recorded only during the last rubbing cycle. The rate of occurrence of these major fluctuations tended to increase during the subsequent rubbing cycles. Simultaneously with the fluctuation, significant drops in the coefficient of friction were detected. Similar sudden major fluctuations were not observed for the SS archwires.

Different explanations have been proposed for the sudden coefficient of friction changes in the available literature. It has been suggested that small metal particles are also detached during the triboelectrochemical experiments, together with the removal of the oxide layer. Such particles might lower the corrosion activity, explaining the electrochemical potential increases and the current drops, simultaneously with decreases in the coefficient of friction [33]. However, this theory does not clarify the differences between the NiTi archwires and the SS archwires, where such fluctuations were not detected. Moreover, the increasing rate of occurrence of the major fluctuations during the rubbing cycles cannot be explained by the spontaneous appearance of a third body. However, it is possible, that persisting residual parts of the oxide layer in notches related to an increased surface roughness, measured especially on NiTi archwires inside the wear track, caused the detected major fluctuations.

Another theory correlates the sudden major fluctuations of the electrochemical current, the potential, and the coefficient of friction to phase transformations of the metal’s crystal structure. It was shown by Razalia et al. [34] that during sliding, the local temperatures of the NiTi archwire increase, and consequently, phase transformations at different depths can be induced. Similarly, Fu et al. [13], using a computer-simulation technique (finite-element analysis), evidenced that rubbing generates more martensite grains in the surface layer of a NiTi archwire. These changes to the metal structure can affect the corrosion and mechanical properties, including the coefficient of friction. In order to verify this hypothesis, another set of experiments using special analytic techniques (i.e., XRD: X-Ray Diffraction) would be needed. However, it should be mentioned that the limited amount of the metal volume (quantity of martensite grains in the overall small volume of the archwires) might make reliable detection difficult.

It is known that another factor that affects the coefficient of friction is surface roughness [35,36]. In accordance with other studies [35,36,37,38], the coefficient of friction for NiTi (both as received and in-vivo aged) was higher than for SS archwires. The surface roughness inside the wear track was substantially higher (3.5 times) for the NiTi archwires than for the SS archwires. It might, therefore, be concluded that the coefficient of friction increase in both the as-received and in-vivo aged NiTi archwires could be related to a slow irreversible phase transformation in the surface layer. This is also in agreement with the higher hardness detected inside the wear track than outside it, especially for the in-vivo aged NiTi archwires. It has recently been reported [39] that the surfaces of orthodontic archwires show a significant degree of variation, and archwires of the same type from the same manufacturer may differ significantly in this respect. Therefore, despite the high accuracy of the in-vitro setting, the sample size examined here-in might be regarded as small, which indeed could be a limitation. Therefore, further research on a larger sample would be needed in order to better elucidate this topic.

## 5. Conclusions

As expected, NiTi archwires showed better repassivation properties and better corrosion resistance than SS archwires since the repassivation time of the SS archwires was significantly longer than that of NiTi archwires wires. Long-term in-vivo archwire exposure decreases their corrosion susceptibility, more evidently for NiTi than SS archwires. However, it should be mentioned that the scatter of the obtained values for the as-received NiTi was significantly higher than the in-vivo exposed NiTi archwires, while the opposite was seen for SS archwires.

The coefficient of friction was lower for the SS archwires than for the NiTi archwires, but the difference was not substantial. In the case of NiTi archwires, the friction coefficients were slightly lower for the in-vivo aged ones, whereas in the case of SS archwires, no evident difference was observed.

Sudden major fluctuations of electrochemical current and potential, which coincided with the drops of the coefficient of friction, were observed on NiTi archwires. Since these fluctuations were more frequent on in-vivo aged than as-received NiTi archwires and their frequency increased at the following rubbing cycles, it is possible that they occurred due to localized residual parts of the oxide layer persisting due to the increased surface roughness or could be a result of phase transformations of the NiTi alloy crystal structure. No similar fluctuations were measured on the SS archwires.

No significant differences in the depth of the tribocorrosion damage on the archwires were found, although the damage on the in-vivo aged NiTi archwire was slightly deeper. Specific parallel scratches were identified on all the types of archwires. Hardness was generally higher inside the tribocorrosion track than outside it, which confirms that the tribological process-induced certain plastic deformation of the alloys.

## Figures and Tables

**Table 1:** Mean values and standard error of the mean [S.E.] with a 95% confidence interval of repassivation times (minutes), electrochemical potentials (mV), currents (nA), and friction coefficient during the triboelectrochemical experiment for as-received and in-vivo aged NiTi and SS archwires.

Parameter	Cycle	NiTi		SS	
		As-Received	In-Vivo Aged	As-Received	In-Vivo Aged
repassivation time					
(minutes)	Cycle 1	72.4 [59.0] ^1^	5.6 [2.7]	279.1 [126.8]	329.1 [308.4]
	Cycle 2	87.2 [64.7]	8.3 [1.5]	378.6 [27.2]	324.1 [253.5]
	Cycle 3	50.8 [29.0]	9.3 [7.4]	431.1 [71.7]	167.8 [150.7]
	Cycle 4	82.9 [60.3]	6.3 [1.2]	402.3 [97.3]	281.0 [192.3]
electrochemical potential					
(mV)	Cycle 1	−200 [167]	−213 [72]	−159 [42]	−184 [158]
	Cycle 2	−232 [197]	−228 [73]	−218 [37]	−228 [134]
	Cycle 3	−244 [179]	−257 [24]	−252 [37]	−263 [138]
	Cycle 4	−226 [110]	−240 [34]	−306 [23]	−264 [93]
electrochemical current					
(nA)	Cycle 1	152 [185]	74 [28]	261 [79]	96 [122]
	Cycle 2	123 [126]	110 [72]	360 [60]	86 [79]
	Cycle 3	107 [85]	83 [21]	385 [6]	157 [87]
	Cycle 4	175 [197]	98 [47]	345 [128]	312 [261]
friction coefficient					
	Cycle 1	0.661 [0.08]	0.638 [0.04]	0.578 [0.01]	0.554 [0.02]
	Cycle 2	0.657 [0.07]	0.634 [0.05]	0.556 [0.04]	0.564 [0.05]
	Cycle 3	0.672 [0.04]	0.624 [0.05]	0.549 [0.04]	0.545 [0.07]
	Cycle 4	0.666 [0.04]	0.668 [0.06]	0.522 [0.03]	0.562 [0.07]

^1^ data of one specimen was excluded since repassivation did not occur after cycle 1.

**Table 2:** Mean and standard error of the mean [S.E.] of roughness (Ra in µm) and hardness outside and inside the wear track of the as-received and in-vivo aged NiTi and SS archwires.

Parameter	Area of the Wear Track	NiTi		SS	
		As-Received	In-Vivo Aged	As-Received	In-Vivo Aged
roughness					
(Ra in µm)	outside	0.3157 [0.068]	0.1567 [0.001]	0.0180 [0.002]	0.5010 [0.114]
	inside	0.0839 [0.010]	0.0938 [0.012]	0.0254 [0.004]	0.0248 [0.010]
hardness					
	outside	210 [25]	150 [30]	445 [15]	450 [225]
	inside	230 [30]	330 [20]	560 [90]	390 [40]

## Data Availability

The data underlying this article will be shared on reasonable request to the corresponding author.

## Supplementary Materials

The following supporting information can be downloaded at: https://www.mdpi.com/article/10.3390/ma15041427/s1, Figure S1: Electrochemical potential (U), current (I), and coefficient of friction measured on three (red, blue, green) as-received NiTi archwires (highlighted sections represent intervals where the specimen was exposed to tribocorrosion cycles); Figure S2: Electrochemical potential (U), current (I), and coefficient of friction measured on three (red, blue, green) in-vivo aged NiTi archwires (highlighted sections represent intervals where the specimen was exposed to tribocorrosion cycles); Figure S3: Electrochemical potential (U), current (I), and coefficient of friction measured on three (red, blue, green) as-received SS archwires (highlighted sections represent intervals where the specimen was exposed to tribocorrosion cycles); Figure S4: Electrochemical potential (U), current (I), and coefficient of friction measured on three (red, blue, green) in-vivo aged SS archwires (highlighted sections represent intervals where the specimen was exposed to tribocorrosion cycles); Figure S5: Working electrode surface (left) and counter electrode surface (right) both obtained from an as-received NiTi archwire with labeled areas of EDS analysis: 1: edge of the wear track, 2: area outside the wear track, 3: area inside the wear track; 4, 5, 6 counter NiTi electrode; Figure S6: Working electrode surface (left) and counter electrode surface (right) both obtained from an in-vivo aged NiTi archwire with labeled areas of EDS analysis: 1: edge of the wear track, 2: area outside the wear track, 3: area inside the wear track; 4, 5 counter NiTi electrode; Figure S7: Working electrode surface (left) and counter electrode surface (right) both obtained from an as-received SS archwire with labeled areas of EDS analysis: 1: edge of the wear track, 2: area outside the wear track, 3: area inside the wear track; 4, 5 counter SS electrode; Figure S8: Working electrode surface (left) and counter electrode surface (right) both obtained from an in-vivo aged SS archwire with labeled areas of EDS analysis: 1: area outside the wear track; 2,3: edge of the wear track; 4,5: area inside the wear track; 6, 7 counter SS electrode; Table S1: EDS surface analysis expressed as weight % of specific chemical elements detected at six locations (shown in Figure S5) of an as-received NiTi archwire; Table S2: EDS surface analysis expressed as weight % of specific chemical elements detected at six locations (shown in Figure S6) of an in-vivo aged NiTi archwire; Table S3: EDS surface analysis expressed as weight % of specific chemical elements detected at six locations (shown in Figure S7) of an as-received SS archwire; Table S4: EDS surface analysis expressed as weight % of specific chemical elements detected at six locations (shown in Figure S8) of an in-vivo aged SS archwire.
