# Supplementary material for: Tribocorrosion Susceptibility and Mechanical Characteristics of As-Received and Long-Term In-Vivo Aged Nickel-Titanium and Stainless-Steel Archwires
Source: Materials (Basel). 2022 Feb 15;15(4):1427. doi: 10.3390/ma15041427 (PMC8876863; doi:10.3390/ma15041427)
Supplement: Supplementary file 1 [file materials-15-01427-s001.zip › materials-1550703-supplementary.pdf]

Supplementary materials

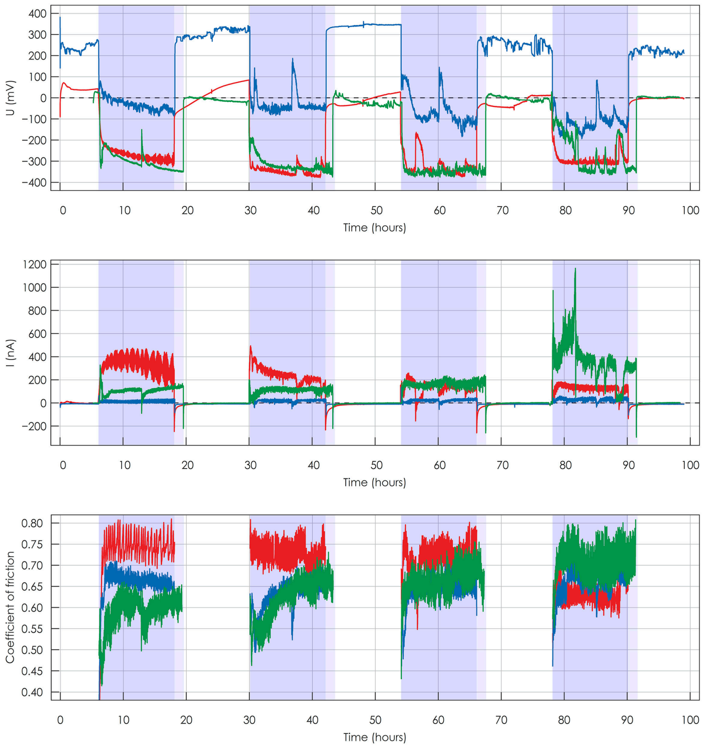

Figure S1. Electrochemical potential (U), current (I), and coefficient of friction measured on three (red, blue, green) as-received NiTi archwires (highlighted sections represent intervals where the specimen was exposed to tribocorrosion cycles).

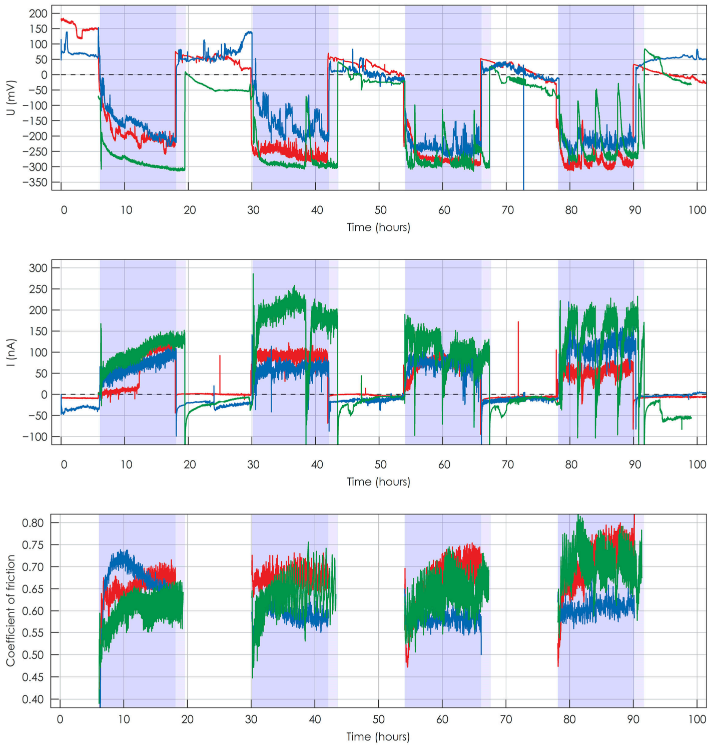

Figure S2. Electrochemical potential (U), current (I), and coefficient of friction measured on three (red, blue, green) in-vivo aged NiTi archwires (highlighted sections represent intervals where the specimen was exposed to tribocorrosion cycles).

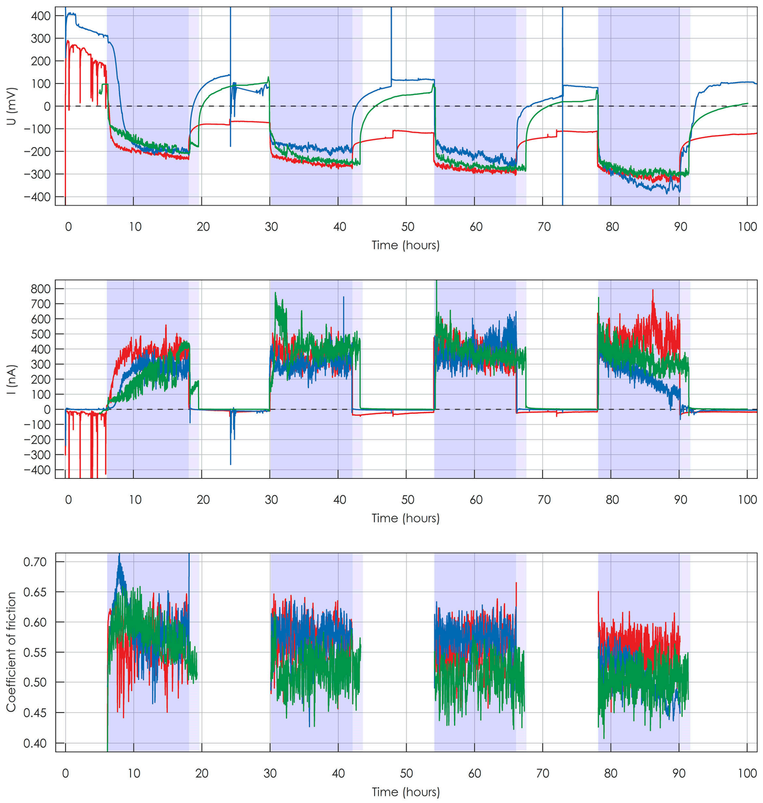

Figure S3. Electrochemical potential (U), current (I), and coefficient of friction measured on three (red, blue, green) as-received SS archwires (highlighted sections represent intervals where the specimen was exposed to tribocorrosion cycles).

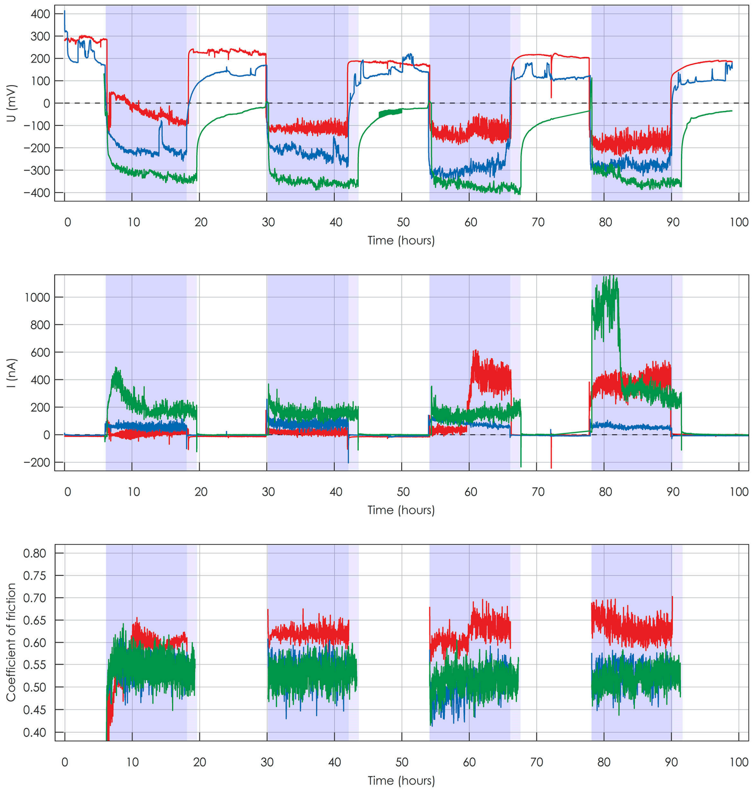

Figure S4. Electrochemical potential (U), current (I), and coefficient of friction measured on three (red, blue, green) in-vivo aged SS archwires (highlighted sections represent intervals where the specimen was exposed to tribocorrosion cycles).

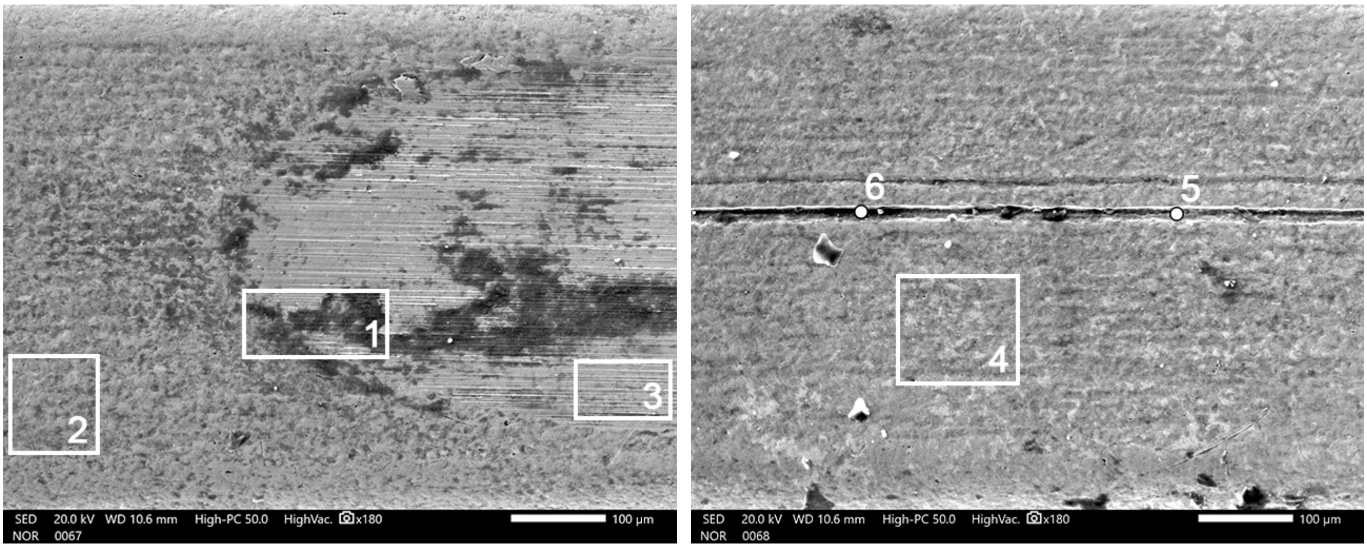

Figure S5. Working electrode surface (left) and counter electrode surface (right) both obtained from an as-received NiTi archwire with labeled areas of EDS analysis: 1: edge of the wear track, 2: area outside the wear track, 3: area inside the wear track; 4, 5, 6 counter NiTi electrode.

Table S1. EDS surface analysis expressed as weight % of specific chemical elements detected at six locations (shown in Figure S5) of an as-received NiTi archwire.

Location	C	O	P	K	Ca	Ti	Ni
**1**	17.24	16.76	1.15	0.47	0.61	30.06	33.72
**2**	10.52	2.75	-	-	-	39.68	47.06
**3**	11.55	4.35	-	-	-	38.38	45.73
**4**	9.99	2.86	-	-	-	39.84	47.31
**5**	12.10	3.04	-	-	-	38.00	46.86
**6**	25.37	5.29	-	-	-	31.15	38.19

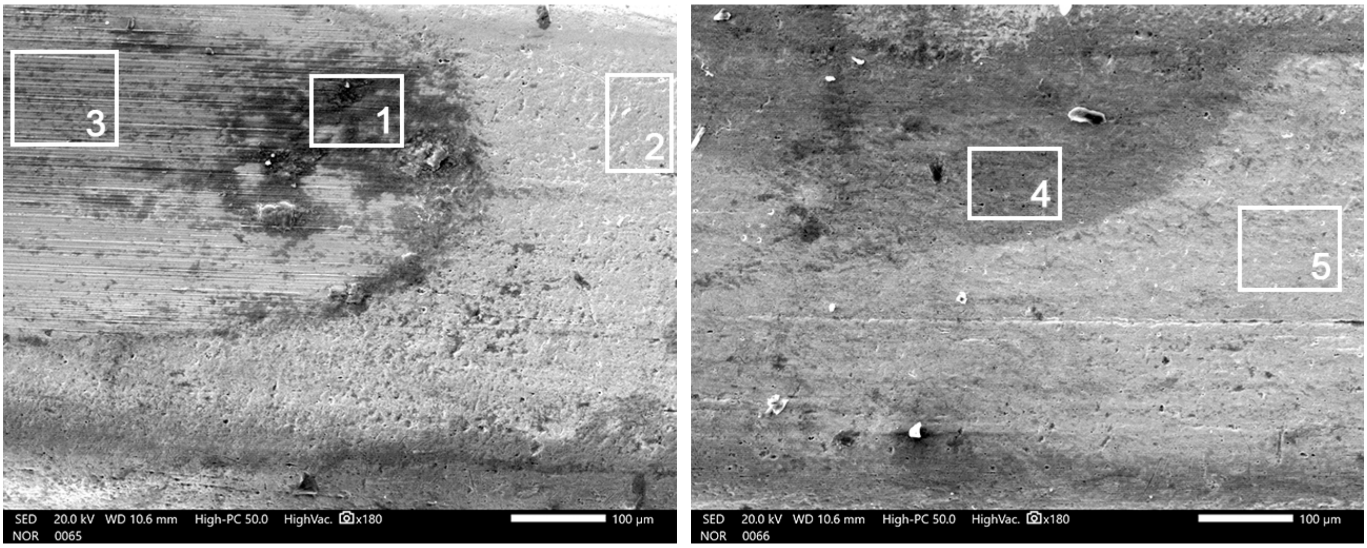

Figure S6. Working electrode surface (left) and counter electrode surface (right) both obtained from an in-vivo aged NiTi archwire with labeled areas of EDS analysis: 1: edge of the wear track, 2: area outside the wear track, 3: area inside the wear track; 4, 5 counter NiTi electrode.

Table S2. EDS surface analysis expressed as weight % of specific chemical elements detected at six locations (shown in Figure S6) of an in-vivo aged NiTi archwire.

Location	C	O	Na	Si	P	K	Ca	Ti	Ni	Al
**1**	19.29	19.37	1.68	0.21	1.3	1.69	0.26	27.34	28.86	-
**2**	8.72	2.48	-	-	-	-	-	40.71	48.09	-
**3**	12.41	5.46	-	-	-	0.18	-	37.58	44.37	-
**4**	12.52	7.38	-	-	0.29	0.17	0.38	36.22	43.03	-
**5**	8.58	3.37	-	-	-	-	0.20	39.81	47.85	0.18

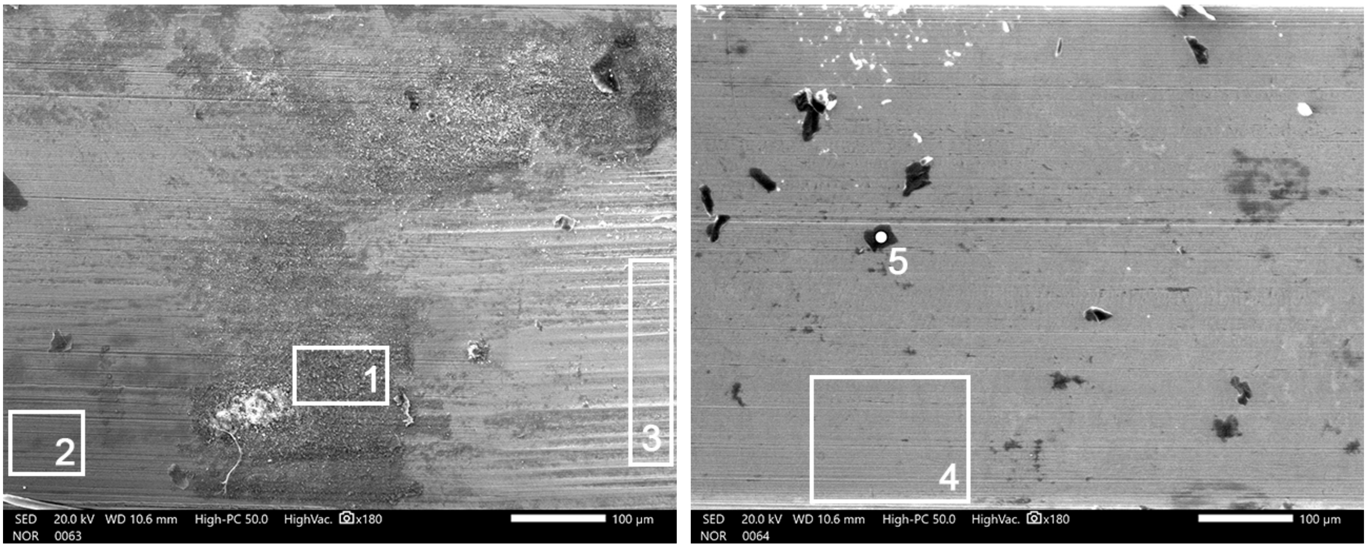

Figure S7. Working electrode surface (left) and counter electrode surface (right) both obtained from an as-received SS archwire with labeled areas of EDS analysis: 1: edge of the wear track, 2: area outside the wear track, 3: area inside the wear track; 4, 5 counter SS electrode.

Table S3. EDS surface analysis expressed as weight % of specific chemical elements detected at six locations (shown in Figure S7) of an as-received SS archwire.

Location	C	O	Na	Al	Si	P	S	Cl	K	Ca	Cr	Mn	Fe	Ni
**1**	15.19	24.55	4.31	0.13	0.31	5.57	0.13	0.90	4.41	1.12	8.49	0.60	31.37	2.92
**2**	9.57	4.27	1.00	0.30	0.61	0.62	-	0.12	0.25	0.16	16.48	1.07	58.31	7.24
**3**	9.61	2.93	0.80	0.33	0.61	0.35	-	-	0.23	-	17.1	1.06	59.66	7.31
**4**	9.75	1.66	-	0.61	0.70	-	-	-	-	-	17.29	1.23	61.21	7.55
**5**	42.60	4.72	-	0.21	0.35	-	0.09	0.10	-	-	10.59	0.69	36.37	4.27

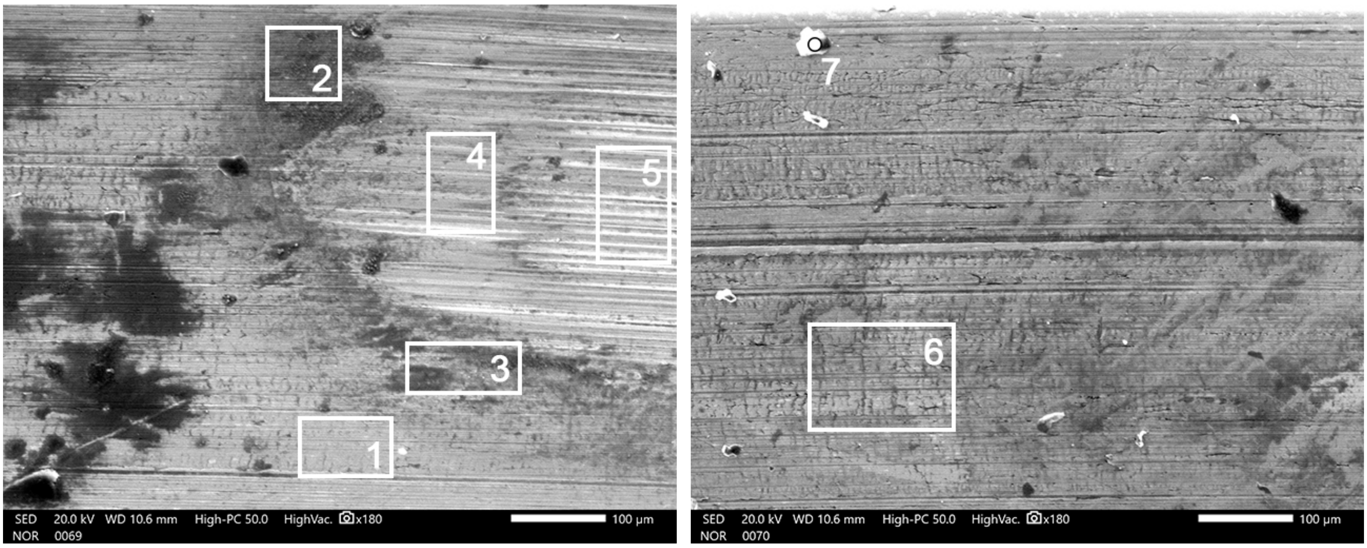

Figure S8. Working electrode surface (left) and counter electrode surface (right) both obtained from an in-vivo aged SS archwire with labeled areas of EDS analysis: 1: area outside the wear track; 2,3: edge of the wear track; 4,5: area inside the wear track; 6, 7 counter SS electrode.

Table S4. EDS surface analysis expressed as weight % of specific chemical elements detected at six locations (shown in Figure S8) of an in-vivo aged SS archwire.

Location	C	O	Na	Al	Si	P	S	Cl	K	Ca	Cr	Mn	Fe	Ni	Mo	N
**1**	10.05	1.74	-	0.17	0.58	0.15	-	-	-	-	16.85	0.99	62.06	6.82	0.59	-
**2**	20.20	9.72	1.14	0.15	0.45	1.26	0.15	0.23	0.45	0.46	12.71	0.71	47.29	5.09	-	-
**3**	17.68	11.85	1.31	0.13	0.42	1.73	0.17	0.26	0.75	0.48	12.6	0.82	46.74	5.05	-	-
**4**	9.98	1.36	-	0.25	0.56	-	-	-	-	-	16.83	0.88	62.59	7.09	0.46	-
**5**	10.73	1.28	-	0.24	0.51	-	-	-	-	-	17.05	0.9	62.11	7.18	-	
**6**	11.64	1.32	-	-	0.51	-	-	-	-	-	16.72	0.87	61.72	6.74	0.48	-
**7**	47.85	8.47	0.17	-	0.26	-	0.21	-	-	0.08	7.41	0.45	25.9	2.61	-	6.58
